# Effects of non-dominant side training on athletic performance: a systematic review

**DOI:** 10.3389/fphys.2025.1602586

**Published:** 2025-07-24

**Authors:** Yugang Zhang, Chengye Jin, Min Sun, Lei Zhang

**Affiliations:** ^1^Department of Physical Education, Yuncheng University, Yuncheng, Shanxi, China; ^2^School of Physical Education, Yeungnam University, Daegu, Republic of Korea; ^3^School of Physical Education, Hangzhou Normal University, Hangzhou, Zhejiang, China

**Keywords:** athlete, non-dominant side, specialized skills, speed, training effects

## Abstract

Evidence suggests that non-dominant side training benefits athletic performance. However, there is a lack of systematic reviews on the effects of non-dominant side training on athletes’ performance. This study aims to synthesize existing research on the impact of non-dominant side training on athletic performance. Following the PRISMA (Preferred Reporting Items for Systematic Reviews and Meta-Analyses) guidelines, a systematic search was conducted on 16 June 2024, across the VIP Database, PubMed, Web of Science Core Collection, EBSCOhost, and CINAHL Plus. The search strategy adhered to the PICOS framework and included a combination of keywords related to non-dominant side training, athletic performance, and experimental studies. After screening 269 identified studies, nine studies were included in this systematic review. The included studies were evaluated using the PEDro scale, with a mean score of 4.22, indicating moderate methodological quality. Analysis of the selected studies revealed that a total of 359 athletes aged 12–24 years participated in non-dominant side training interventions. The intervention durations ranged from 8 weeks to 16 months, with training frequencies varying from once to four times per week. The findings demonstrated significant effects of non-dominant side training on athletic technique, strength, change-of-direction speed, jumping ability, balance, and mental rotation tasks. Furthermore, the results indicate that non-dominant side training has a significant impact across different sports disciplines and age groups. These findings provide evidence-based training strategies to optimize athletic performance in various sports.

**Systematic Review Registration:**

https://www.crd.york.ac.uk/prospero, Identifier CRD42024551710.

## Introduction

Achieving excellence in competitive sports is often attributed to a specialized training process (Smith, 2003). Evaluating effective training methods and models can provide valuable insights for coaches and physical educators, helping athletes and students enhance motor skills (Jiménez-Díaz et al., 2019). It is well established that technical proficiency and motor skills are closely related to athletic performance. In a competitive match, performance is not determined by a single technical skill but rather by a comprehensive set of technical movements (Majumdar and Robergs, 2011). For instance, in tennis, performance is not solely dependent on serving, forehand, or backhand strokes, but rather on the combined execution of multiple tennis techniques (Macun and Söğüt, 2024). Therefore, enhancing athletes’ overall motor skills through effective training methods is crucial for improving competitive performance.

With the advancement of sports science and technology, modern coaches and athletes are increasingly recognizing the importance of developing and maintaining well-rounded motor skills (Siekańska et al., 2021). Various training methods have been investigated, including plyometric training (Deng et al., 2023), eccentric training (Hou and Wang, 2023), altitude training (Silva, 2024), and non-dominant side training (Haaland and Hoff, 2003). Notably, non-dominant side training (NDST) is a training strategy that focuses on enhancing the non-dominant side of athletes (Lipecki, 2017). This approach aims to balance and strengthen an athlete’s dominant and non-dominant sides, improving overall athletic performance and reducing injury risk (Bigoni et al., 2017). In competitive sports, coaches often incorporate non-dominant side training to enhance athletes’ performance (Haaland and Hoff, 2003) and reduce injury risk by improving strength and balance on the non-dominant side (Lanshammar and Ribom, 2011). In specific sports, basketball coaches implement NDST to improve jump height and jump rate (Kim et al., 2013), while soccer coaches use it to enhance lower-limb strength and balance, thereby reducing injuries (Svensson et al., 2018).

Previous research has extensively explored the physiological and mechanical mechanisms underlying non-dominant side training and its effects on athletic performance (Wang and Fu, 2019; Kim et al., 2013). In sports education and competitive training, the key benefits of non-dominant side training include but are not limited to:(a) Injury prevention (Svensson et al., 2018), (b) Enhanced jumping ability (Kim et al., 2013), (c) Improved athletic performance (Turkova et al., 2021), and (d) Increased bone mineral density (BMD) (van Santen et al., 2019). Additionally, in basketball skill acquisition, NDST enhances limb coordination and facilitates skill application, leading to more efficient learning (Tousi et al., 2017). In judo, higher proportions of non-dominant side training improve movement coordination, increase lateral movement speed, and enhance technical execution in live combat (Iglesias-Soler et al., 2018). Based on the above analysis, NDST appears to improve various physical characteristics, supporting the hypothesis that non-dominant side training can enhance technical skill performance in athletes.

A literature review revealed that only one systematic review has examined the differences between unilateral training (dominant side training) and bilateral training (training both dominant and non-dominant sides) in motor skill development (Zhang et al., 2023). Most studies have focused on balance ability (Bettariga et al., 2022) and overall health outcomes (Thompson and Watson, 2019). A comprehensive review of existing literature suggests that the impact of non-dominant side training on technical and skill performance remains unclear. Meanwhile, an increasing number of experimental studies have explored the effects of NDST on specific skills, such as golf—where enhancing non-dominant side strength has been shown to improve driving performance (Sung et al., 2016). However, these findings lack systematic compilation. Therefore, this systematic review aims to synthesize existing empirical research on NDST and evaluate its effectiveness in enhancing athletic performance. By elucidating its applicability across diverse populations and training contexts, the study seeks to provide a robust theoretical foundation and practical implications for future sports training practices. Furthermore, a critical analysis of research methodologies is conducted to highlight current limitations and outline directions for future research.

## Methods

### Protocol and registration

The selection, collection, and analysis of data in this review were conducted in accordance with the Preferred Reporting Items for Systematic Reviews and Meta-Analyses (PRISMA) guidelines and were prospectively registered at PROSPERO (www.crd.york.ac.uk/prospero/) under the registration number CRD42024551710.

### Search strategy

**FIGURE 1:**
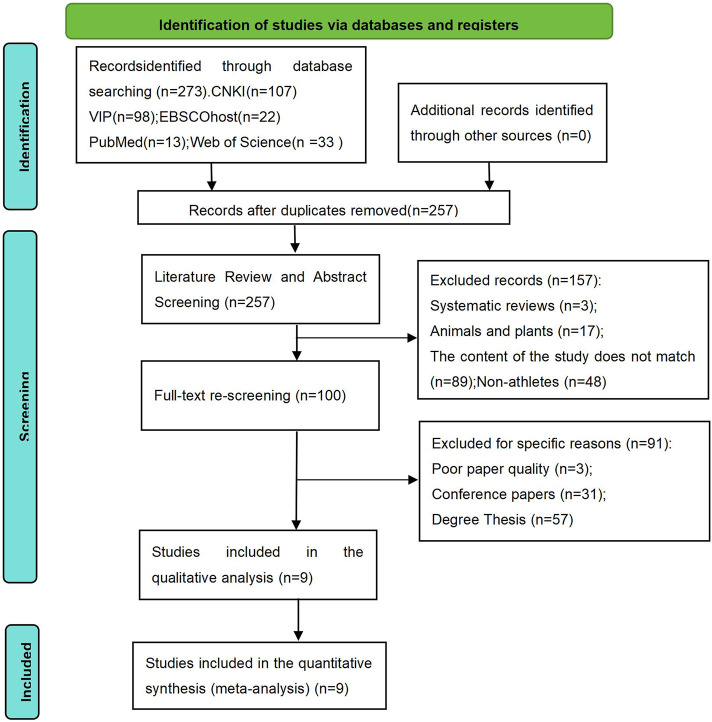
PRISMA flow diagram.

On 16 June 2024, a comprehensive search was conducted across five electronic databases to retrieve relevant literature on this topic: China National Knowledge Infrastructure (CNKI), VIP Database, PubMed, Web of Science, and EBSCOhost (SPORTDiscus). The search strategy was developed based on previous systematic reviews (Zhang et al., 2023; Bettariga et al., 2022; Thompson and Watson, 2019) and the terminology used in related studies. A keyword-based search strategy combined with Boolean operators was applied across all five databases. The search was conducted using the following keywords and Boolean operators: “Non-dominant side” OR “Non-dominant limb” OR “Non-dominant hand” OR “Non-dominant arm” OR “Non-dominant leg” OR “Non-dominant foot” OR “Non-preferred side” OR “Non-preferred limb” AND “athletic performance” OR “technical skill” OR “skill” OR “technique” OR “performance” AND “athlete*” OR “player*”. Additionally, to prevent data omission, a manual search was performed in Google Scholar to supplement the retrieved materials. The literature selection process involved four main stages (Figure 1):Removing duplicate records. Excluding studies written in languages other than Chinese and English. Screening study titles and abstracts to remove systematic reviews, studies unrelated to athletes, and non-human (plant/animal) studies. Full-text screening, during which conference papers, dissertations, and low-quality studies were excluded. The literature selection process was independently conducted by ZYG and SM. In cases of disagreement, discussions were held to reach a consensus. If necessary, a third reviewer (ZL) was involved in the decision-making process until a final agreement was reached.

### Eligibility criteria

The eligibility assessment of the included studies was conducted using the PICOS framework (Population, Intervention, Comparison, Outcomes, and Study Design). The inclusion criteria were as follows:

Population: The study participants were athletes, with no restrictions on gender, age, or sport discipline.

Intervention: The minimum duration of non-dominant side training (NDST) was 4 weeks. The training program could focus on the upper limbs, lower limbs, or both. The exercises included non-dominant side technical training, strength training, speed training, or a combination of these, typically involving pre-stretching or reverse motion loading on the non-dominant side.

Control: The control group followed a traditional training program without non-dominant side training interventions.

Outcomes: The included studies had to report at least one assessment of the effects of non-dominant side training on athletes’ technical skill performance. Additionally, the studies needed to provide pre- and post-intervention performance measurements of the experimental subjects in technical skill tests.

Study Design: Only randomized controlled trials (RCTs) were considered in this systematic review.

Studies that did not meet these inclusion criteria were excluded.

### Data extraction and quality assessment

The two authors (ZYG and ZL) systematically extracted and recorded information from the included studies using Microsoft Excel spreadsheets. To ensure data accuracy, a third author (ZL) conducted an independent review of the recorded information.

The extracted study information included the following elements:

a) First author’s name and year of publication.
b) Characteristics of the study participants, including age, gender, sport discipline, and sample size.
c) Details of the non-dominant side training intervention, including training duration, training intensity, and control group intervention.
d) Pre- and post-training assessment indicators of the participants.
e) Evaluation metrics for both the non-dominant side training group and the control group.

The two authors (ZYG and ZL) independently assessed the methodological quality of the included studies using the PEDro scale, a tool designed for grading the methodological quality of research. The PEDro scale has demonstrated high reliability (Appleby et al., 2020) and validity (Sung et al., 2016) in previous studies. To ensure consistency and accuracy, a third author (ZL) conducted a discrepancy check between the two assessments. Any disagreements were resolved through discussion until a consensus was reached among all three authors. The PEDro scale consists of 11 evaluation criteria, each scoring one point. However, the first item (external validity criterion) is excluded from the total score, resulting in a maximum possible score of 10 points. The quality assessment interpretation based on PEDro scores is as follows:1–3 points: Low methodological quality, 4–5 points: Moderate methodological quality 6–10 points: High methodological quality. The items included in the scale used in this study are presented in Table 1.

**TABLE 1:** Summary of methodological quality assessment scores.

Study	Haaland and Hoff. (2003)	Lu et al. (2016)	Sung et al.(2016)	Ramirez-Campillo et al. (2018)	Witkowski et al. (2011)	Ben Kahla et al. (2022)	Zhang et al.(2024)	Pietsch and Jansen (2018)	Guilherme et al. (2015)	Total
Eligibility Criteria	1	1	1	1	1	1	1	1	1	9
Random Allocation	1	1	1	1	1	1	1	1	1	9
Allocation Concealment	0	0	0	0	0	0	0	0	0	0
Baseline Comparability	1	1	1	1	1	1	1	1	1	9
Blind Participants	1	0	0	0	0	0	0	0	0	1
Blind Therapist	0	0	0	0	0	0	0	0	0	0
Blind Assessor	1	0	0	0	0	0	0	0	0	1
Follow- Up	0	0	0	0	0	0	0	0	0	0
Intention to Treat Analysis	0	0	0	0	0	0	0	0	0	0
Between Group Comparisons	1	1	1	1	1	1	1	1	1	9
Point Measure and Variability	1	1	1	1	1	1	1	1	1	9
Total PEDro Score	6	4	4	4	4	4	4	4	4	

## Results

Based on a thorough screening and review of the retrieved literature, this systematic review includes nine studies—comprising both RCTs and non-RCTs—that examine the effects of non-dominant limb training on athletic performance. These studies were published between 2003 and 2022. The characteristics of the included studies are summarized in Table 2.

**TABLE 2:** Characteristics of the studies examined in the present review.

Study	Population	Intervention						Main outcome related to skills
	N	Type of athletes	Gender	Age	Type	Skill measured index	Frequency & duration	
Haaland and Hoff. (2003)	39	soccer player	Male	EG1:15 years EG2:17 years EG1:Above 19 years old CG1:16 years CG2:18 years CG3p: Above 19 years old	EG: Non-dominant side soccer skill training CG: Conventional soccer skill training	Foot-Tapping Test; Shooting Accuracy; Passing Accuracy; (Dribbling/Agility;	8weeks	Foot-Tapping Test↑; Shooting Accuracy↑; Passing Accuracy↑; Dribbling/Agility↑
Lu et al. (2019)	14	badminton player	Male	12 ± 1 year	EG:Non-dominant side strength training + badminton skill training CG:Conventional strength training + badminton skill training	Bilateral Muscle Strength; Strength Endurance; Flexor-Extensor Torque Balance; Sustained Lower Limb Loading; Maximal Strength and Agility;	40 weeks, twice per week, 90 min per session	Bilateral Muscle Strength↑; Strength Endurance↑; Flexor-Extensor Torque Balance↑; Sustained Lower Limb Loading↑; Maximal Strength and Agility↑;
Sung et al.(2016)	60	golf player	Male	CEG:23.0 ± 0.5 years NCEG:23.2 ± 0.6 years CG:24.0 ± 1.0 years	EG: Conventional training NCEG: Core muscle training + non-dominant arm training CG: Conventional Training	Drive Distance; Trunk Strength; Non-Dominant Arm Strength; Wrist Extension; Elbow Extension;	8 weeks, six times per week, 60 min per session	Drive Distance↑; Trunk Strength↑; Non-Dominant Arm Strength↑; Wrist Extension↔; Elbow Extension↔
Ramirez-Campillo et al.(2018)	18	soccer player	Male	EG:17.3 ± 1.1 years CG:17.6 ± 0.5 years	EG: Plyometric Training + Non-Dominant Side Strength Training CG: Traditional Training	Knee Extensor Strength (1RM_KE); Knee Flexor Strength (1RM_KF); Change of Direction Ability (COD); Vertical Jump Performance; Limb Symmetry Index (LSI);	8weeks, 4 sessions per week	Knee Extensor Strength (1RM_KE)↑; Knee Flexor Strength (1RM_KF)↑; Change of Direction Ability (COD)↑; Vertical Jump Performance↑; Limb Symmetry Index (LSI)↑;
Witkowski et al. (2011)	47	soccer player	Male	13 years	EG:Traditional Training Model + Bilateral Training (Equal Duration and Intensity Ratio of 1:1 for Dominant and Non-Dominant Sides) CG:Traditional Training Model with Asymmetrical Bilateral Training (Duration and Intensity Ratio of 4:1 for Dominant and Non-Dominant Sides)	Dribbling Efficiency; Effectiveness of Aerial Ball Control; Effectiveness of Short-Distance Inside-Foot Passing in the Air; Effectiveness of Long-Distance Inside-Foot Passing in the Air; Effectiveness of Short-Distance Inside-Foot Passing on the Ground; Effectiveness of Long-Distance Inside-Foot Passing on the Ground;	1 year, 3 sessions per week, 96 min per session	Dribbling Efficiency↑; Effectiveness of Aerial Ball Control↑; Effectiveness of Short-Distance Inside-Foot Passing in the Air↑; Effectiveness of Long-Distance Inside-Foot Passing in the Air↑; Effectiveness of Short-Distance Inside-Foot Passing on the Ground↑; Effectiveness of Long-Distance Inside-Foot Passing on the Ground↑;
Ben Kahla et al. (2022)	60	soccer player	Male	15.8 ± 0.6 years	EG:Dominant-Side and Non-Dominant-Side Training CG:Traditional Skill Training	Running Agility; Skill; Dribbing; Passing; Shooting;	12 weeks,4 sessions per week, 72 min per session	Running Agility↑; Skill↑; Dribbing↑; Passing↑; Shooting↑;
Zhang et al.(2024)	30	Basketball Player	Male	EG:20.9 ± 1.1year CG:20.9 ± 0.9years	EG:Unilateral Combined Training (Resistance Training and Plyometric Training) with a Dominant-to-Non-Dominant Side Ratio of 1:3 CG:Conventional Training	Body Morphology; Single-Leg Countermovement Jump (SLCMJ); Isometric Mid-Thigh Pull Test (IMTP); Countermovement Jump (CMJ); Standing Long Jump (SLJ); Squat Performance;	10 weeks,4 sessions per week, including 3 sport-specific training sessions and 1 physical conditioning session per week	Body Morphology↑; Single-Leg Countermovement Jump (SLCMJ)↑; Isometric Mid-Thigh Pull Test (IMTP)↑; Countermovement Jump (CMJ)↑; Standing Long Jump (SLJ)↑; Squat Performance↑;
Pietsch and Jansen (2018)	20	soccer player	19 males and 1 female	EG:10.6 ± 0.5 years CG:10.5 ± 0.5 years	EG:Non-Dominant-Side Sport-Specific Skill Training CG:Conventional Sport-Specific Skill Training	Mental Rotation Task; Shoot Test; Dribbling Test; Ball Control;	10 weeks,1 session per week	Mental Rotation Task↑; Shoot Test↑; Dribbling Test↔; Ball Control↔;
Guilherme et al. (2015)	71	soccer player	Male	EG1:12.17 ± 0.72 years EG2:14.36 ± 0.5 years EG3:16.58 ± 0.51 year CG1:12.42 ± 0.67 years CG2:14.50 ± 0.52 years CG3:16.58 ± 0.61 year	EG:Non-Dominant-Side Sport-Specific Skill Training CG:Conventional Sport-Specific Skill Training	Motor Skills of the Non-Dominant Leg; Utilization Rate of the Non-Dominant Leg in Competition;	16 months, U13and U15 trained 3 times per week, while U17 trained 4 times per week	Motor Skills of the Non-Dominant Leg↑; Utilization Rate of the Non-Dominant Leg in Competition↑;

EG, Experimental Group; CG, Control Group; ↑, The experimental effect is significant; ↔, No significant effect was observed.

### Study selection

The selection process of the included studies followed four distinct phases (Figure 1).

First, a total of 273 potential studies were identified through the systematic search strategy, with 13 from PubMed, 22 from EBSCOhost, 107 from CINAHL Plus, 33 from Web of Science, and 98 from VIP database. After removing 16 duplicate records, the remaining articles proceeded to the next phase.

Second, a preliminary screening was conducted based on title and abstract. A total of 157 studies were excluded due to the following reasons: animal and plant research, systematic reviews, irrelevant study content, or non-athlete populations. Third, full-text screening was performed to assess study quality, resulting in the exclusion of 91 studies, including low-quality papers, dissertations, and conference proceedings. Finally, 9 studies met all inclusion criteria and were included in the final analysis. Throughout the selection process, two authors (ZYG and SM) independently screened the articles. In cases of disagreement, a third author (ZL) reviewed the disputed studies, and consensus was reached through discussion.

### Study quality assessment

Any discrepancies encountered during the screening process were resolved through discussion. If necessary, a third author (ZL) participated in the verification and decision-making process until a consensus was reached.

Table 1 presents the scoring details of the PEDro scale for the included studies. The average score of the included studies was 4.22 (range: 3–4), indicating a moderate quality of the studies. Since the included studies focused on exercise training interventions, there was a potential risk of injury during the training process. Consequently, the included studies were penalized in the areas of allocation concealment, blinding of therapists, and intention-to-treat (ITT) analysis. Among the included studies, only Haaland and Hoff (2003) implemented blinding of participants and blinding of assessors. The highest compliance with the PEDro scale was observed in eligibility criteria (n = 9), random allocation (n = 9), between-group comparisons (n = 9), and point measures and variability (n = 9).

### Study characteristics

Table 1 presents the characteristics of participants and interventions in the included RCT studies. Between 2003 and 2024, all eligible Chinese and English-language studies meeting the inclusion criteria were reviewed. Among the nine included studies, seven employed a two-arm experimental design (Haaland and Hoff, 2003; Lu et al., 2016; Ramirez-Campillo et al., 2018; Ben Kahla et al., 2022; Zhang et al., 2024; Pietsch and Jansen, 2018; Gstöttner et al., 2009), while two studies adopted a three-arm design (Sung et al., 2016; Witkowski et al., 2011). Regarding the sport-specific focus of the studies, among the nine included studies:One study examined basketball players (Zhang et al., 2024). Six studies focused on soccer players (Haaland and Hoff, 2003; Ramirez-Campillo et al., 2018; Witkowski et al., 2011; Ben Kahla et al., 2022; Pietsch and Jansen, 2018; Gstöttner et al., 2009). One study investigated badminton players (Lu et al., 2016). One study focused on golf players (Sung et al., 2016). A total of 359 athletes participated in the included studies, with seven studies specifically involving youth athletes (Haaland and Hoff, 2003; Lu et al., 2016; Ramirez-Campillo et al., 2018; Witkowski et al., 2011; Ben Kahla et al., 2022; Guilherme et al., 2015).

Regarding the intervention duration: Three studies conducted an 8-week intervention (Sung et al., 2016; Haaland and Hoff, 2003; Witkowski et al., 2011). Two studies conducted a 10-week intervention (Zhang et al., 2024; Pietsch and Jansen, 2018). One study conducted a 12-week intervention (Ben Kahla et al., 2022). One study conducted a 40-week intervention (Lu et al., 2016). Two studies implemented interventions lasting 1 year or longer (Witkowski et al., 2011; Guilherme et al., 2015). In terms of comparison groups, among the nine studies: Three studies incorporated non-dominant side strength training and sport-specific skill training as comparative interventions (Lu et al., 2016; Sung et al., 2016; Ramirez-Campillo et al., 2018). Six studies used non-dominant side sport-specific skill training as a comparative intervention (Haaland and Hoff, 2003; Witkowski et al., 2011; Ben Kahla et al., 2022; Zhang et al., 2024; Pietsch and Jansen, 2018; Guilherme et al., 2015).

### Effects of non-dominant side training on athletic performance

The study conducted an analysis of nine selected outcomes, demonstrating the effects of non-dominant side training on athletes in terms of sports techniques, strength, jumping ability, and psychological attributes. Table 2 summarizes the key findings from the nine included studies.

### Effects of non-dominant side training on athletes’ technical performance

Among the nine included studies, six investigated the effects of non-dominant side training on sport-specific technical performance (Sung et al., 2016; Haaland and Hoff, 2003; Witkowski et al., 2011; Ben Kahla et al., 2022; Pietsch and Jansen, 2018; Guilherme et al., 2015). Of these six studies, five focused on adolescent athletes (Haaland and Hoff, 2003; Witkowski et al., 2011; Ben Kahla et al., 2022; Pietsch and Jansen, 2018; Guilherme et al., 2015), while one examined adult athletes (Sung et al., 2016). In total, these six studies involved 295 athletes, among whom one was female. One study (Sung et al., 2016) investigated improvements in golf technical performance, with driving distance as the primary outcome measure. The other five studies focused on technical performance in soccer players (Haaland and Hoff, 2003; Witkowski et al., 2011; Ben Kahla et al., 2022; Pietsch and Jansen, 2018; Guilherme et al., 2015), with the following specific metrics: Four studies assessed dribbling ability (Haaland and Hoff, 2003; Witkowski et al., 2011; Ben Kahla et al., 2022; Pietsch and Jansen, 2018). Two studies evaluated agility (Haaland and Hoff, 2003; Pietsch and Jansen, 2018). Three studies measured shooting accuracy (Haaland and Hoff, 2003; Pietsch and Jansen, 2018; Ben Kahla et al., 2022). Two studies analyzed passing accuracy (Haaland and Hoff, 2003; Witkowski et al., 2011). Two studies examined the utilization rate of the non-dominant leg (Pietsch and Jansen, 2018; Guilherme et al., 2015).

### Effects of non-dominant side training on athletes’ strength

Among the nine included studies, three analyzed the effects of non-dominant side training on athletes’ strength (Sung et al., 2016; Lu et al., 2016; Ramirez-Campillo et al., 2018). A total of 92 male athletes participated in these trials. Among them, two studies focused on adolescent athletes (Lu et al., 2019; Ramirez-Campillo et al., 2018), while one study examined adult athletes (Sung et al., 2016). Regarding the sports disciplines: One study involved badminton players (Lu et al., 2016). One study focused on golfers (Sung et al., 2016). One study analyzed soccer players (Ramirez-Campillo et al., 2018). In terms of strength assessment: Two studies used flexor-extensor muscle strength as the primary outcome measure (Lu et al., 2019; Ramirez-Campillo et al., 2018). One study assessed core muscle strength (Sung et al., 2016).

### Effects of non-dominant side training on athletes’ change of direction speed

Among the nine included studies, two analyzed the effects of non-dominant side training on athletes’ change of direction speed (Haaland and Hoff, 2003; Ramirez-Campillo et al., 2018). A total of 57 male athletes participated in these trials, all of whom were adolescent athletes. Both studies focused on soccer players (Haaland and Hoff, 2003; Ramirez-Campillo et al., 2018). Regarding the assessment metrics: One study used the foot-tap test as the primary measure (Haaland and Hoff, 2003). One study assessed change of direction (COD) ability (Ramirez-Campillo et al., 2018).

### Effects of non-dominant side training on athletes’ jumping performance

Among the nine included studies, two analyzed the effects of non-dominant side training on athletes’ jumping performance (Zhang et al., 2024; Ramirez-Campillo et al., 2018). A total of 48 male athletes participated in these trials. One study focused on soccer players (Ramirez-Campillo et al., 2018), while the other examined basketball players (Zhang et al., 2024). One study investigated adolescent athletes (Ramirez-Campillo et al., 2018), whereas the other focused on adult athletes (Zhang et al., 2024). Regarding the jump performance assessment: One study measured vertical jump performance (Ramirez-Campillo et al., 2018). One study evaluated single-leg countermovement jump (SLCMJ), bilateral countermovement jump (CMJ), and standing long jump (SLJ) (Zhang et al., 2024).

### Effects of non-dominant side training on athletes’ balance ability

Among the nine included studies, two examined the effects of non-dominant side training on athletes’ balance ability (Lu et al., 2019; Ramirez-Campillo et al., 2018). A total of 32 male athletes participated in these trials. One study focused on badminton players (Lu et al., 2019), while the other investigated soccer players (Ramirez-Campillo et al., 2018). Both studies targeted adolescent athletes (Lu et al., 2019; Ramirez-Campillo et al., 2018). Regarding balance assessment: One study used flexor-extensor torque balance as the primary outcome measure (Lu et al., 2019). One study assessed limb symmetry index (LSI) (Ramirez-Campillo et al., 2018).

### Effects of non-dominant side training on athletes’ mental rotation task performance

Among the nine included studies, in addition to the effects of non-dominant side training on athletes’ explicit motor performance, one study reported its impact on athletes’ mental rotation task ability (Pietsch and Jansen, 2018). This study involved 20 adolescent soccer players, including 19 males and 1 female. The primary outcome measure was the Mental Rotation Task (MRT). Regarding the intervention:

The experimental group underwent conventional sport-specific skill training combined with non-dominant side skill training. The control group received only conventional sport-specific skill training.

## Discussion

This study presents a comprehensive review of the effects of non-dominant side training on athletes’ performance. The primary objective is to collect and analyze existing research, synthesize academic findings, identify research trends and consensus, and provide a foundation for future studies and training methodologies related to non-dominant side training.

The findings indicate that non-dominant side training contributes to improvements in technical performance, strength, jumping ability, and psychological attributes in athletes. However, several factors may influence the experimental outcomes, including intervention frequency and duration, age, sport type, and gender. Despite these variables, existing studies have demonstrated positive effects. Nevertheless, can non-dominant side training serve as an effective training strategy to enhance athletic performance? Based on the findings presented in the Results section, this review provides a detailed analysis of the various dimensions influencing athletes’ performance.

### Overview of experimental design for non-dominant side training

On ball sports with distinct dominant and non-dominant side differences. The biomechanical distinctions between the dominant and non-dominant sides in these sports align with competitive characteristics, facilitating data collection and information analysis (Pollard et al., 2020), thus providing a solid foundation for experimental research. However, the same intervention may yield different effects across various training programs (Andreoli et al., 2001). Differentiating gender, age, and sports discipline in experimental comparisons enhances the reliability of the results (Bornstein, 2011). However, in the included studies, researchers primarily employed mixed experimental and control group designs, without strictly controlling for gender differences. Notably, one study even included both male and female athletes within the same experimental group (Pietsch and Jansen, 2018). Given that non-dominant side training may affect athletic performance differently based on gender, the lack of gender-specific analysis imposes certain limitations on the applicability of the experimental findings.

The effectiveness of exercise intervention research is influenced by multiple factors, among which frequency, session count, and intervention duration are key variables. Different experimental studies have shown varying effects of intervention frequency, session count, and duration on outcomes (Bailey and Brooke-Wavell, 2010). Among the nine studies included in this review, the shortest intervention lasted 8 weeks, while the longest extended to 16 months. The variation in intervention duration may be associated with expected training outcomes and differences in study populations (Johnson et al., 2014; Bherer et al., 2005). For strength training interventions, the American College of Sports Medicine (ACSM) recommends performing at least two training sessions per week for major muscle groups, with 1–3 sets per session (Thompson et al., 2013). Consequently, the experimental designs in this review primarily focus on studies involving strength training, sport-specific skill development, and athletes’ psychological wellbeing. To expand the applicability of non-dominant side training, future research should explore its effects on additional aspects of athletic performance beyond the current study scope.

Raining intervention design is a core component of sports science experiments, significantly influencing scientific validity, data reliability, and the generalizability of conclusions (Atkinson and Nevill, 2001). Among the nine studies included in this review, the experimental designs assessing the same athletic performance outcome incorporated various training strategies in combination with non-dominant side training. These interventions produced differing effects on athletes within the same sport, along with unintended outcomes beyond the primary measurement parameters. In two studies, researchers employed different training ratios between the dominant and non-dominant sides as intervention variables, which led to both similar and distinct effects on athletes within the same sport (Lu et al., 2019; Ramirez-Campillo et al., 2018). This suggests that non-dominant side training can exert variable intensities and multifaceted influences on athletic performance.

Issues in Measurement Tool Selection. Among the nine studies included in this review, researchers employed different testing methods to assess the same performance metric. For instance, in agility testing, researchers adapted measurement protocols based on sport-specific skill characteristics, such as those of soccer (Haaland and Hoff, 2003) and badminton (Lu et al., 2019). Similarly, inconsistencies were observed in dribbling performance assessment for soccer players. Witkowski et al. (2011) conducted assessments based on prior research methodologies, whereas Pietsch and Jansen (2018) adhered to the standardized guidelines of the German Research Association. The lack of standardized measurement tools in experimental research may compromise the accuracy of findings (Subramaniam and Silverman, 2000). Although the nine included studies confirmed the positive impact of non-dominant side training on athletic performance, limitations in experimental design highlight areas for improvement. Future research should not only account for individual differences among participants but also further refine intervention protocols and establish standardized measurement tools to enhance the applicability of non-dominant side training in athletic performance development.

### Effects of non-dominant side training on athletes’ technical performance

Existing literature provides substantial evidence supporting the advantages of non-dominant side training over traditional training methods in enhancing sport-specific skills. Technical performance in athletes refers to the mastery and execution of movement patterns and techniques in a specific sport (Newell, 2020). It reflects an athlete’s proficiency and application of sport-specific techniques. Among the nine studies included in this review, six studies reported a significant impact of non-dominant side training on athletes’ technical performance (Sung et al., 2016; Haaland and Hoff, 2003; Witkowski et al., 2011; Ben Kahla et al., 2022; Pietsch and Jansen, 2018; Guilherme et al., 2015). These studies demonstrated that non-dominant side training significantly improved the following technical skills: Dribbling, ball control, and passing ability in soccer players (Haaland and Hoff, 2003; Witkowski et al., 2011; Ben Kahla et al., 2022; Pietsch and Jansen, 2018; Guilherme et al., 2015). Driving distance in golf players (Sung et al., 2016). Beyond elite sports, non-dominant side training has also shown positive effects in physical education, contributing to the improvement of basketball skills among university students (Duan et al., 2024). However, it is important to note that among the six studies included in this analysis, all subjects were male athletes, primarily adolescents, and only soccer and golf players were investigated. This limited the generalizability of the findings across different sports, genders, and age groups. Furthermore, all included studies implemented a hybrid training approach, combining non-dominant side training with other training methods. However, the intervention duration varied considerably, ranging from 8 weeks to 16 months, which may have influenced the consistency of the findings. Future Research Directions To improve the applicability of non-dominant side training, future research should: Expand the study population to include athletes of different genders, age groups, and sports disciplines. Investigate the effects of non-dominant side training across different stages of skill development, further enriching the scientific understanding of its role in athletic performance. Standardize intervention protocols, particularly in terms of training duration and intensity, to enhance the reliability and applicability of research outcomes.

### Effects of non-dominant side training on athletes’ muscular strength

In most sports, athletes tend to rely on their dominant side to perform strength-based movements, such as shooting in soccer, spiking in volleyball, and shooting in basketball. However, non-dominant side strength training also plays a crucial role in overall athletic performance. Training the non-dominant side can enhance core strength and coordination, contributing to more balanced and comprehensive physical development. Previous research has demonstrated that targeted non-dominant side training can improve overall strength and body control (Munn et al., 2004). Among the nine studies included in this review, three investigated the effects of non-dominant side strength training on muscle strength adaptations (Sung et al., 2016; Lu et al., 2016; Ramirez-Campillo et al., 2018). All three studies established measurement criteria based on the technical characteristics of the respective sports. Additionally, one study specifically examined the effects of non-dominant side strength training on core muscle strength (Sung et al., 2016). Findings from these studies suggest that combined strength training, which integrates non-dominant side training, is more effective than conventional training in enhancing muscular strength. However, some studies indicate that in female athletes, non-dominant side strength training may influence strength development in the dominant side differently compared to traditional strength training (Abazović, Kovačević, Kovač and Bradić, 2015). Other research suggests that non-dominant side strength training activates a greater number of core muscles to maintain stability, thereby improving overall athletic performance (Cirer-Sastre et al., 2017). Implications and Future Research Directions these findings highlight that non-dominant side strength training, or its integration into mixed training programs, has a positive impact on muscular strength development in athletes. However, it is important to note that the studies included in this review focused only on muscle groups directly related to the athletes’ sports and did not comprehensively examine the broader impact of non-dominant side training on overall strength development. Additionally, limitations in sample selection may have affected the generalizability of the results. Future research should involve larger sample sizes, with particular emphasis on female athletes and participants from diverse age groups, in order to enhance the applicability and generalizability of the findings. Conduct systematic investigations into the effects of non-dominant side strength training on various muscle groups and performance metrics. Expand the application of non-dominant side training beyond current limitations to optimize its effectiveness in athletic training programs.

### Effects of non-dominant side training on athletes’ change of direction speed

Among the nine studies included in this review, two studies examined the effects of non-dominant side training on athletes’ change of direction (COD) ability (Haaland and Hoff, 2003; Ramirez-Campillo et al., 2018). Change of direction ability refers to an athlete’s capacity to efficiently and rapidly alter movement direction (Falch et al., 2019). In these two studies, researchers employed different assessment methods: The Foot-Tapping Test was used in one study (Haaland and Hoff, 2003). The Change of Direction (COD) test was utilized in the other (Ramirez-Campillo et al., 2018). The findings provide valuable insights for coaches seeking to enhance athletes’ change of direction ability. However, the limited sample size constrains the practical implications of these findings. It is important to note that multiple factors influence an athlete’s change of direction ability, including muscle strength, perception-decision making, and technique (Dos Santos et al., 2017). A study on change of direction speed and technique enhancement demonstrated that technical interventions improved athletes’ COD speed (Dos Santos et al., 2022). To further validate the effects of non-dominant side training on change of direction speed, future research should: Incorporate key influencing factors, such as strength, decision-making, and biomechanics, to develop a more comprehensive understanding. To improve the reliability and external applicability of research findings, future studies should include larger sample sizes to examine the effects of non-dominant side training across different genders and age groups. In addition, the design of training protocols should be expanded to further explore its sport-specific adaptability within targeted athletic disciplines.

### Effects of non-dominant side training on athletes’ jumping performance

Existing literature provides evidence supporting the advantages of mixed training methods incorporating non-dominant side training over traditional training approaches in improving athletes’ jumping performance. Jumping performance refers to an athlete’s ability to generate explosive force against the ground, which primarily depends on muscular strength, power, neuromuscular coordination, and technique (Stojanović et al., 2017). Jumping ability is crucial in sports such as basketball, volleyball, track and field, and soccer. Among the nine studies included in this review, two studies reported a significant impact of mixed non-dominant side training on athletes’ jumping performance (Lu et al., 2019; Ramirez-Campillo et al., 2018). The effectiveness of mixed training methods that integrate non-dominant side training in enhancing jumping ability has garnered considerable research interest. Yanci and Camara (2016) found that compared to traditional training methods, mixed non-dominant side training led to significant improvements in vertical jump performance among soccer players. Unlike previous studies, this review did not focus exclusively on the relationship between non-dominant side training and jumping performance. However, the findings contribute to the broader understanding of how mixed non-dominant side training influences multiple aspects of athletic performance rather than just a single dimension. It is noteworthy that the two included studies primarily assessed vertical jump, standing long jump, and single- and double-leg jump performance. However, the potential impact of non-dominant side training on reactive jump performance and approach jump ability remains unexplored and warrants further investigation in future research.

### Effects of non-dominant side training on athletes’ balance ability

Balance ability refers to an athlete’s capacity to maintain postural stability, adjust the center of gravity, and rapidly regain balance in both static and dynamic conditions. It is a fundamental prerequisite for executing technical movements in competition and a key determinant of success in sports performance (Ricotti, 2011). Existing literature provides substantial evidence supporting the advantages of mixed non-dominant side training over traditional training methods in enhancing athletes’ balance ability. Among the nine studies included in this review, two studies (Lu et al., 2019; Ramirez-Campillo et al., 2018) examined the effects of mixed non-dominant side training on postural stability, reporting significant improvements compared to traditional training approaches. These studies assessed balance performance using: Flexor-extensor torque balance (Lu et al., 2019). Limb symmetry index (LSI) (Ramirez-Campillo et al., 2018). The experimental design in these studies deviated from conventional training methods, which traditionally emphasize the dominant side. Instead, researchers increased the training duration and frequency for the non-dominant side, leading to greater muscular strength development and improved motor control in the non-dominant limb. These findings highlight the role of non-dominant side training in enhancing postural stability and provide valuable insights for coaches seeking to improve balance ability in athletes. Notably, the included studies assessed balance ability without integrating technical skill execution, leaving uncertainty regarding the impact of non-dominant side training on sport-specific balance performance. Future research should: Investigate whether non-dominant side training enhances balance ability during sport-specific movements. Explore the interaction between non-dominant side strength training and motor skill acquisition. Expanding both sample size and training protocols is essential to enhance the applicability of research findings across various sports disciplines and among populations differentiated by gender and age. Furthermore, standardizing intervention procedures and systematically evaluating the effects of varying intervention intensities across groups will help to clarify the practical value of non-dominant side training.

### Effects of non-dominant side training on athletes’ mental rotation task performance

Sports training not only influences athletes’ physical fitness and motor skills but also has a significant impact on mental rotation ability. Existing research suggests that systematic athletic training can enhance psychological resilience, improve emotional regulation, and alleviate competition-related anxiety (Chung et al., 2013). However, previous studies have primarily focused on the psychological effects of general physical activity (Husain et al., 2024). Among the nine studies included in this review, only one study reported a significant effect of mixed non-dominant side training on athletes’ mental rotation task performance compared to traditional training methods (Pietsch and Jansen, 2018). This study employed the mental rotation task (MRT) scale developed by Vandenberg and Kuse (1978), providing valuable insights into the broader impact of non-dominant side training on athletic performance. It is important to note that mental rotation task performance varies based on gender and profession (Fargier et al., 2022). However, whether non-dominant side training differentially affects mental rotation ability across genders or specific sports disciplines remains unclear. This gap in the current research limits the generalizability of findings. Investigate gender-specific and sport-specific effects of non-dominant side training on mental rotation performance. Increasing the sample size and standardizing assessment tools are critical for enhancing the applicability of findings across different genders and age groups, as well as extending their relevance to a broader range of sports disciplines. Further explore the cognitive benefits of non-dominant side training to optimize its application in athletic performance enhancement.

## Limitations

Although this review provides evidence supporting the effects of non-dominant side training on athletes’ skill performance, several limitations were identified, summarized as follows:

1) The number of included studies was relatively small, and the number of participating athletes was limited. Most participants were male athletes, indicating a lack of gender diversity, which constrains the generalizability of findings. In addition, the age range of participants was broad (10–24 years), including pubertal, post-pubertal, and young adult males. This heterogeneity in physical and physiological development may affect the interpretation of intervention outcomes.
2) Existing studies primarily focused on strength training and sport-specific skill training in relation to NDST. There is a lack of research on other forms of NDST, such as agility or coordination training, or its integration with multiple training modalities, which limits a comprehensive understanding of its effects.
3) The duration of NDST interventions varied considerably, ranging from 8 weeks to 16 months, making it difficult to compare outcomes across studies. Furthermore, the included studies only covered four types of sports, restricting the applicability of findings. Future research should investigate the effects of NDST across a wider range of sports disciplines to enhance its relevance and practical utility.

## Conclusion

Our findings suggest that non-dominant side training can enhance athletic performance to some extent. Notably, non-dominant side training has been shown to improve: Sport-specific skills (soccer players, golfers) Muscular strength (badminton players, soccer players) Jumping ability (soccer players, basketball players) Balance ability (soccer players).

Mental rotation ability (soccer players) However, given the limitations identified in this review, it is essential to interpret these findings with caution. To better understand the effectiveness of non-dominant side training in enhancingathletic performance, comprehensive research is required, taking into account differences in gender and sports disciplines. Furthermore, future studies should adopt a multidisciplinary and cross-disciplinary approach to explore the impact of non-dominant side training on athletes’ body composition and physiological adaptations.

## Data Availability

The original contributions presented in the study are included in the article/supplementary material, further inquiries can be directed to the corresponding author.
